# Spatial variability and environmental drivers of cassava—arbuscular mycorrhiza fungi (AMF) associations across Southern Nigeria

**DOI:** 10.1007/s00572-021-01058-x

**Published:** 2022-01-04

**Authors:** Bolaji Thanni, Roel Merckx, Pieterjan De Bauw, Margaux Boeraeve, Gerrit Peeters, Stefan Hauser, Olivier Honnay

**Affiliations:** 1 Division Soil and Water Management, Department of Earth and Environmental Sciences, KU Leuven, Kasteelpark Arenberg 20, box-3001, 3001 Heverlee, Leuven, Belgium; 2 Department of Biology, Plant Conservation and Population Biology, KU Leuven, B-3001 Leuven, Belgium; 3 Root and Tuber Agronomy, International Institute of Tropical Agriculture, Ibadan, Nigeria

**Keywords:** Cassava, Arbuscular mycorrhizal fungi, Biodiversity, Next-generation sequencing, Agricultural practices, Soil chemical properties

## Abstract

Cassava, forming starch-rich, tuberous roots, is an important staple crop in smallholder farming systems in sub-Saharan Africa. Its relatively good tolerance to drought and nutrient-poor soils may be partly attributed to the crop’s association with arbuscular mycorrhiza fungi (AMF). Yet insights into AMF-community composition and richness of cassava, and knowledge of its environmental drivers are still limited. Here, we sampled 60 cassava fields across three major cassava-growing agro-ecological zones in Nigeria and used a DNA meta-barcoding approach to quantify large-scale spatial variation and evaluate the effects of soil characteristics and common agricultural practices on AMF community composition, richness and Shannon diversity. We identified 515 AMF operational taxonomic units (OTUs), dominated by *Glomus*, with large variation across agro-ecological zones, and with soil pH explaining most of the variation in AMF community composition. High levels of soil available phosphorus reduced OTU richness without affecting Shannon diversity. Long fallow periods (> 5 years) reduced AMF richness compared with short fallows, whereas both zero tillage and tractor tillage reduced AMF diversity compared with hoe tillage. This study reveals that the symbiotic relationship between cassava and AMF is strongly influenced by soil characteristics and agricultural management and that it is possible to adjust cassava cultivation practices to modify AMF diversity and community structure.

**Graphical abstract:**

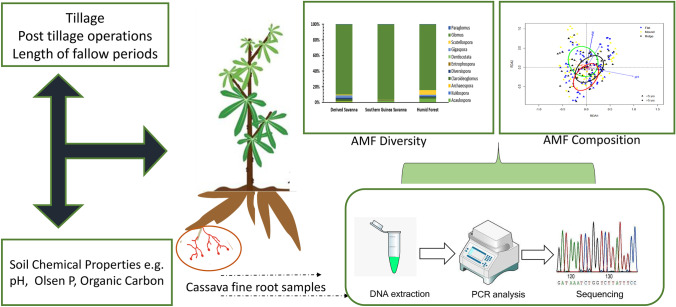

**Supplementary information:**

The online version contains supplementary material available at 10.1007/s00572-021-01058-x.

## Introduction

Cassava (*Manihot esculenta*) is a perennial woody shrub native to South America (Li and Zhu 2011; Okudoh et al. 2014), forming tuberous roots rich in starch. The introduction of the crop to sub-Saharan Africa (SSA) started in the sixteenth century through Portuguese traders (Okigbo 1980). Due to the hardy nature of the crop, it is well adapted to the farming conditions and systems of many smallholder farmers in SSA. Currently, 40 African countries account for 61% of global cassava production (Ezedinma 2017) and roughly 40% of Africans consume it as a dietary staple (Nweke et al. 2002). With the expected increase in human population in SSA, estimated to reach 2 billion by 2050, meeting the increasing demand for food through sustainable agricultural systems forms a huge challenge. Cassava is a promising crop in this context based on its ability to grow on relatively marginal soils where other crops do not produce at all (Kuiper et al. 2007) and on the possibility of its year-round root supply. The latter has an obvious food security benefit but also offers interesting commercial opportunities for the cassava processing industry.

Cassava has been labelled as the ‘drought, war and famine crop’ partly due to the crop’s ability to withstand drought (El-Sharkawy 2004). Although cassava yields decline appreciably during periods of drought (Oyetunji et al. 2007; Vandegeer et al. 2013), the relatively good tolerance of cassava to drought may be partly attributed to the crop’s association with and reliance on arbuscular mycorrhizal fungi (AMF) (Renker et al. 2005; Rodriguez and Sanders 2015). AMF (*Glomeromycota*) are root symbionts, commonly present in agricultural soils and associated with about 71% of all plant species (Brundrett and Tedersoo 2018). Apart from alleviating drought stress and nutrient deficiency in the host plant (Augé 2001; Birhane et al. 2012; Ceballos et al. 2013; Aliyu et al. 2019) by forming a hyphal network extending widely through the soil (Bowles et al. 2016), they also are beneficial to overall soil quality (Rillig et al. 2019) by contributing to soil aggregation (Barbosa et al. 2019), via the release of glomalin (Liang et al. 2015) and hyphae extension that entangles soil particles (Rillig et al. 2015).

AMF community composition is known to vary across local to regional and continental scales (Öpik et al. 2013; Davison et al. 2016). Such spatial patterns may result from environmental heterogeneity due to differences in soil and vegetation, either natural or because of human management, and from dispersal limitation of propagules of different AMF taxa (Wolfe et al. 2007; Paz et al. 2021; Xu et al. 2014). Yet, especially in Africa, there is a paucity of information on local and regional patterns of AMF community composition, particularly in important crop species such as cassava. So far, the few studies on cassava were conducted at very narrow geographical scales and they relied largely on the visual identification of AMF taxa through spores (Angelov et al. 1993; Oyetunji et al. 2007; Aliyu et al. 2019). Nevertheless, understanding the drivers of variation in AMF community composition of crops is important because it may affect the host plants’ performance. This may be due to continuing co-selection or assembly processes between various plant species and the microbial communities in their rhizospheres (Hahl et al. 2020). For example, a meta-analysis based on greenhouse inoculation studies reported that specific matches between AMF genera and crop host plant families were more beneficial for growth promotion than other combinations (Van Geel et al. 2016). Apart from AMF characteristics, other variables such as host plant features and soil nutrient status can modify plant responses to AMF (Wehner et al. 2010; Lin et al. 2015).

In Nigeria, land preparation methods for cassava cultivation are diverse; some farmers plant the crop on fields with shortened fallow periods usually dominated by grasses and broadleaved weeds, while others plant after longer fallow periods on fields with bush regrowth and forest cover. The cropping phase varies from 1 to 5 consecutive years. Due to the differences in the floristic composition of the planted fields caused by the fallow length, practices to eliminate the natural vegetation (weeds, bushes and trees) also differ. Short-fallow vegetation is usually slashed with a cutlass, while older fallows require more labour to manually slash the vegetation but leave behind larger amounts of biomass. In both systems, the slashed biomass can either be removed from the field, left to decompose or be burned on the field. Soil management practices (SMP), which include tillage, are an integral part of most agro-ecosystems in SSA and mainly aim to improve crop productivity. In Nigeria, however, the method of tillage mostly depends on the economic status of the farmer. Most resource-poor farmers plant directly using zero or minimal tillage, loosening only the spot where they insert the planting stakes. Where labour is available, mounds or ridges are formed with hand hoes. This is largely limited to small field sizes and is not feasible in large fields. The average area of cassava fields in southwestern and southeastern Nigeria is 0.65 and 0.75 ha per farmer, respectively (Ojiako et al. 2018; Awotide et al. 2019), except for commercial farmers. Most commercial farmers (91%) use mechanized primary tillage, usually, tractor-drawn disc ploughs (Magani and Shave 2011), and 9% proceed to secondary tillage which includes the use of tractor-drawn implements such as harrows and ridgers (Osun State Information Services 2005).

Mechanized tillage disrupts the contact between plant roots and fungi by drastically affecting hyphal survival and proliferation (Kabir 2005; Ingleby et al. 2007). In contrast, zero tillage does not invert the soil and has been shown to promote AMF richness and hyphal networks in the soil (Kabir 2005; Dai et al. 2015). Due to differences in hyphal growth patterns, AMF taxa have been reported to differ in their ability to cope with soil perturbation (Kabir 2011). Because AMF are obligate biotrophs (Smith and Read 2008), long fallow periods also may affect AMF-crop interactions (Lekberg and Koide 2005; Ryan and Graham 2018). Additional soil characteristics, especially nutrient availability and pH, are well known to affect AMF diversity and community composition because they directly affect intraradical colonization and arbuscule development (Lekberg and Koide 2005; Van Geel et al. 2017).

In this study, we evaluated the AMF community composition and diversity in 60 cassava fields across three major agro-ecological zones in Nigeria, covering the states with the largest cassava production, using high throughput sequencing of a fragment of the small subunit (SSU) rRNA gene. Our specific research objectives were to (i) characterize AMF community composition and variability across the major cassava-growing agro-ecological zones in Nigeria, (ii) identify the local environmental drivers of cassava AMF diversity and community composition and (iii) quantify the response of AMF community composition and diversity of cassava to common agronomic practices.

## Material and methods

### Sampling sites and methods

This study was conducted in the states Oyo, Ogun, Osun, Edo, Benue and Cross Rivers, all major cassava-growing states in Nigeria (Fig. S1, Supplementary Information). The six states cover three agro-ecological zones (i.e. humid forest, HF; derived savanna, DS; southern Guinea savanna, SGS) (Table S1). The sampling survey was conducted between July and September 2019 across 13 local government areas (LGAs) (Fig. S1; Table S1). Cassava in these zones was planted in fields close to farmers’ homesteads or distant on most soils without any form of fertilizer. Distant fields mostly were cultivated on large expanses of land interspersed by forest. In the study area, the farmers used different methods of land preparation ranging from simple hand tools to tractors and combinations of both.

In all the states, regardless of the agroecology, land clearing depends on the age and prevailing type of vegetation of the fallow. Fields dominated by woody perennial shrubs and trees usually are manually slashed and felled with cutlasses and sometimes chain saws. The biomass is large and the easiest way of clearing the land is by burning. In young fallows dominated by grasses and broadleaved weeds, the vegetation is either slashed or treated with herbicides (Table S1). Depending on the amount of biomass, the land either is cleared by removing the slash from the field or by burning it. Leaving the biomass to decompose or incorporating it during tillage is uncommon. The commonly planted cassava cultivars vary across the states—about 20 cultivars (improved and local) were mentioned to be grown by the farmers of our study (Table S1). In places where improved cultivars were grown, preference was based on ability to produce high yields and tolerance against pests and diseases. Farmers who planted local cultivars believed their roots contained more starch and less water than improved cultivars.

In total, we sampled 60 fields and sampled 4 cassava plants in each field. The cassavas were 1 m apart from each other and plant sampling was diagonal across rows, totalling 240 samples. The sampled cassava plants were carefully uprooted at ca. 12 to 14 weeks after planting, and from each plant 15 to 20 fine roots ranging from 1 to 3 cm long with a diameter < 2 mm were detached from different positions of the root system and stored to dry using silica gel. A soil sample was collected from 0–20 cm depth in each location where a plant was uprooted, making a total of 240 soil samples for soil chemical analysis (pH, Olsen P, total nitrogen and organic carbon). To capture the local soil management practices (SMPs), a survey was carried out in all sites by administering questionnaires to farmers. The SMPs were categorized by type of tillage: (1) ploughing by tractor, (2) hand hoe tillage and (3) no-till; and by the type of soil shaping before planting: (1) ridging, (2) mounding and (3) planting on flat. Finally, the previous fallow lengths were classified into (1) less or (2) more than 5 years. The classification for the fallow period was based on the criterion of 5 years because the majority of farmers were only able to provide the approximate fallow period, not its specific length.

### Soil chemical analyses

Soil pH was quantified using a glass electrode in a 1:10 soil–water mixture. Olsen P was determined colorimetrically using a Lambda 25 spectrophotometer (Perkin Elmer, Waltham) after shaking 5 g dry soil for 30 min with 0.5 M sodium bicarbonate at pH 8.5. Colour development of the extracts was done using the molybdenum blue method (Robertson et al. 1999). Organic carbon and total nitrogen were determined using a CN analyser (Carlo Erba EA1108), quantifying these two elements after dry combustion.

### Root DNA extraction, PCR amplification and sequencing

Total genomic DNA was extracted from 70 mg dried roots from pooled root samples per plant using the Soil DNA Isolation Plus Kit (Norgen Biotek, Thorold, ON, Canada) according to the manufacturer’s instructions. The 18S SSU rDNA region was amplified using the AMF-specific AMV4.5NF-AMDGR primer pair (Sato et al. 2005; Van Geel et al. 2014). The primer pair was adapted for use in a dual-index sequencing strategy on Illumina MiSeq (Kozich et al. 2013). The PCR mixture comprised 1 µL of extracted DNA, 0.5 µL of each 20 µM primer, 5 µL ALLin HiFi Buffer, 0.3 µL ALLin HiFi DNA Polymerase (2 u/µL (highQu)) and 17.7 µL of water making up a total volume of 25 µL. The PCR, performed on a Bio-Rad T100 thermal cycler (Bio-Rad Laboratories, CA, USA), started with an initial denaturation step at 95 °C for 1 min, followed by 30 cycles of 15 s denaturing for 95 °C, 53 °C for 15 s for annealing and 30 s elongation at 72 °C. The final extension was for 11 s at 72 °C. After purification of the PCR products using Agencourt AMPure XP (Beckman Coulter Life Sciences, Indianapolis, IN, USA), the concentration was measured on a Qubit fluorimeter with the Qubit dsDNA HS assay kit (both from Invitrogen, Carlsbad, CA, USA). Samples were equimolarly pooled and the fragment size of the pooled library was checked with gel electrophoresis. Finally, DNA fragments of the right size (ca. 350 bp) were cut from the gel and purified using the QIAquick Gel Extraction Kit (QIAgen). Sequencing was done on the Illumina MiSeq sequencing platform with the v2 500 cycle reagent kit (Illumina, San Diego, CA, USA) at the Genomics Core facility (Leuven, Belgium).

### Bioinformatics

The demultiplexed sequencing data were processed using USEARCH following the recommended pipeline (Edgar 2013). The pipeline included the following steps: (1) merging forward and reverse reads using the ‘*fastq_mergepairs*’ to form consensus sequences; (2) ensuring correct orientation of the sequences using the ‘*orient*’ command against the MaarjAM database (Öpik et al. 2010); (3) quality filtering the reads with the ‘*fastq_filter*’ command, allowing a maximum expected error of 1.0 for the individual sequences with truncation length set to 200 bp; (4) clustering the sequences into operational taxonomic units (OTUs) at sequence similarity threshold of 97% and removing chimeric OTUs with the ‘*cluster_otus*’ command; (5) finally, using the ‘*otutab*’ command to map each sequence to an OTU and a sample, resulting in an OTU table.

To remove possible erroneous sequences produced during PCR or sequencing (Alberdi et al. 2018), an extra filtering step was included: OTUs that represented fewer than 0.01% of the sequences in a sample were discarded from that sample. For assigning taxonomy, the consensus sequence of each OTU was BLASTed against the Maarj*AM* database (Öpik et al. 2010) and NCBI GenBank with the following criteria for a hit: sequence similarity at least 97%, alignment at least 95% of the shorter sequence, BLAST E‐value < 1^e−50^.

### Data analysis and statistics

Statistical analyses were done in R, version 4.0.2 (R Development Core Team 2012). The R package ‘ggplot2’ was used for graphical presentations (Wickham 2016). Initially, rarefaction curves were computed with the extracted OTUs to validate sequencing quality and to exclude samples that would not provide a reliable representation of what was present in the sequenced samples using the *rarecurve* command in the {*vegan*} package (Fig. S2, Supplementary Information). The AMF richness was determined as the number of OTUs present in a sample, using the *specnumber* function from the {*vegan*} package (Oksanen et al. 2019), while the Shannon diversity index was quantified by calculating the Hill number of order 1 (the exponential of Shannon entropy, *q* = 1).

Soil chemical variables, AMF richness and Shannon diversity were first compared across the three agro-ecological zones, using linear mixed models with ‘field’ as the random factor. The estimated marginal means were calculated using the *emmeans* function in the *emmeans* package from the models, and mean comparisons were carried out using the Tukey HSD post hoc test. Large-scale geographical variation was further explored by using a Mantel test, correlating the matrices with geographic distances among samples and their AMF community similarities based on Bray–Curtis distance, using the {*vegan*} package (Oksanen et al. 2019). We then performed an indicator species analysis to determine the OTUs indicative of the different tillage and soil pH, using the function *multipatt* in the *indicspecies* package (De Caceres et al. 2010).

The soil chemical variables (pH, Olsen P, total N and organic C) and soil management practices (type of tillage, soil shaping and length of fallow period) were modelled as fixed variables against AMF richness using generalized linear mixed models (GLMM) with Poisson distribution, while for Exp(H’) a linear mixed model (LMM) was used. Exp(H’) was log-transformed to achieve a normal distribution. Both GLMM and LMM were performed in the {*lme4*} package in R, and field was always used as a random factor.

A canonical redundancy analysis (RDA) was performed on the Hellinger-transformed OTU table with explanatory variables: pH, Olsen P, total N, organic C, type of tillage, soil shaping and length of fallow period. The best RDA model was selected based on adjusted *R*^2^ with forward selection, using the *ordistep* function in {*vegan*}. The significance of the explanatory variables in the model was determined using the *anova.cca* function with permutation tests with 1000 permutations. Finally, variation partitioning was performed as proposed by Legendre (2008), using the *varpart* function of the R package {*vegan*}, to visualize the overlap in explained variation in AMF composition by using three groups of variables: soil chemical variables, soil management practices and the geographical distances.

## Results

### Variation in soil chemical properties across agro-ecological zones

We observed significant differences in chemical soil properties among the three agro-ecological zones (Table S2). The soils in the humid forest zone were strongly acidic (pH = 4.80 ± 0.04), while soils in the southern Guinea savanna (pH = 5.18 ± 0.09) and derived savanna (pH = 6.05 ± 0.08) were moderately and slightly acidic. Except in the humid forest, organic carbon and total nitrogen values were extremely low, pointing at serious soil degradation (Table S2).

### AMF communities in cassava roots

**Fig. 1:**
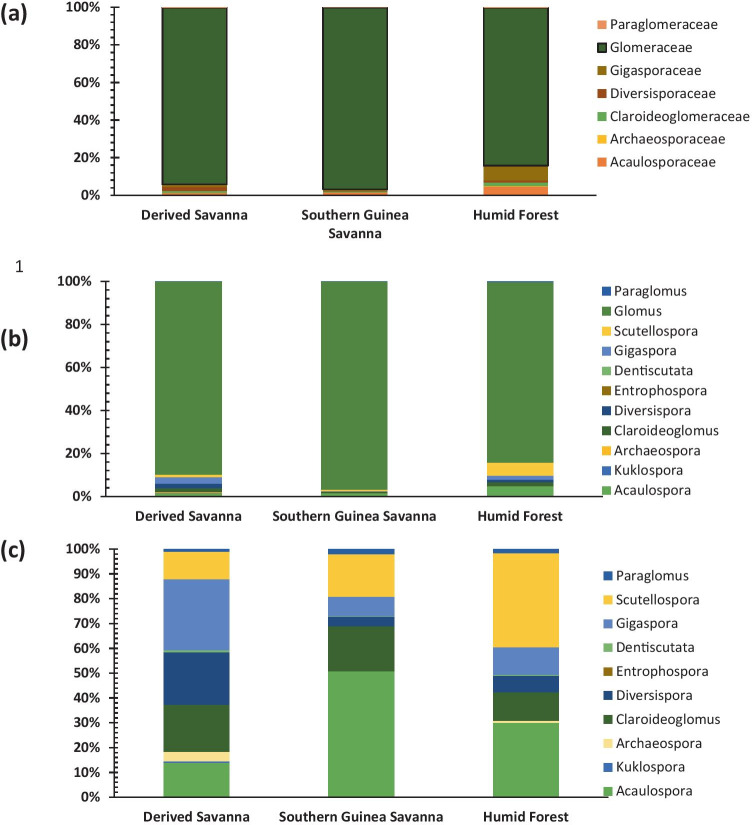
Relative abundances of AMF in the roots of cassava in three agroecology zones in Nigeria. (**a**) Relative read abundance (%) of AMF families present in cassava root samples. (**b**) Relative read abundance (%) of AMF genera present in cassava root samples. (**c**) Relative read abundance (%) of AMF genera present in cassava root samples excluding the genus *Glomus*

Amplification of the 18S SSU rDNA region failed for eight cassava root samples, but a total of 4,390,014 sequences were successfully recovered from 232 root samples after Illumina sequencing. Rarefaction curves (Fig. S2a) indicated that 23 root samples, with fewer than 80 AMF sequences per sample, were insufficiently deeply sequenced to provide a reliable representation of what likely was present in the sample. Removal of these samples led to retaining 209 root samples and 4,380,744 AMF sequences (Fig. S2b). These sequences were allocated to 515 OTUs. Seven AMF families were identified: *Glomeraceae* (78%, 401 OTUs), Claroideoglomeraceae (6%, 29 OTUs), Paraglomeraceae (1%, 7 OTUs), Diversisporaceae (5%, 24 OTU), Acaulosporaceae (4%, 23 OTUs), Gigasporaceae (5%, 25 OTUs) and Archaeosporaceae (1%, 6 OTUs) (Fig. 1a). The seven identified families were present in all the agro-ecological zones but with different levels of dominance. For instance, the Archaeosporaceae and Gigasporaceae were abundant in the humid forest zone while Glomeraceae dominated in all three agro-ecological zones (Fig. 1a). Eleven AMF genera were identified (Fig. 1b) and altogether, the most dominant genus is *Glomus*. When considering the other genera excluding *Glomus* (Fig. 1c), the genera *Scutellospora*, *Gigaspora* and *Acaulospora* were found dominating the humid forest, derived savanna and southern Guinea savanna, respectively, while *Entrophospora* was only present in the derived savanna. The number of OTUs per agro-ecological zone, however, did not differ significantly (Table S2).

### Large-scale spatial variability in AMF communities

The geographic distances between the sampled agro-ecological zones significantly affected AMF community dissimilarity. The communities became more dissimilar as the geographic distance between sites increased, notwithstanding high unexplained variation (*r* = 0.12, *p* = 0.001) (Fig. S3).

**Fig. 2:**
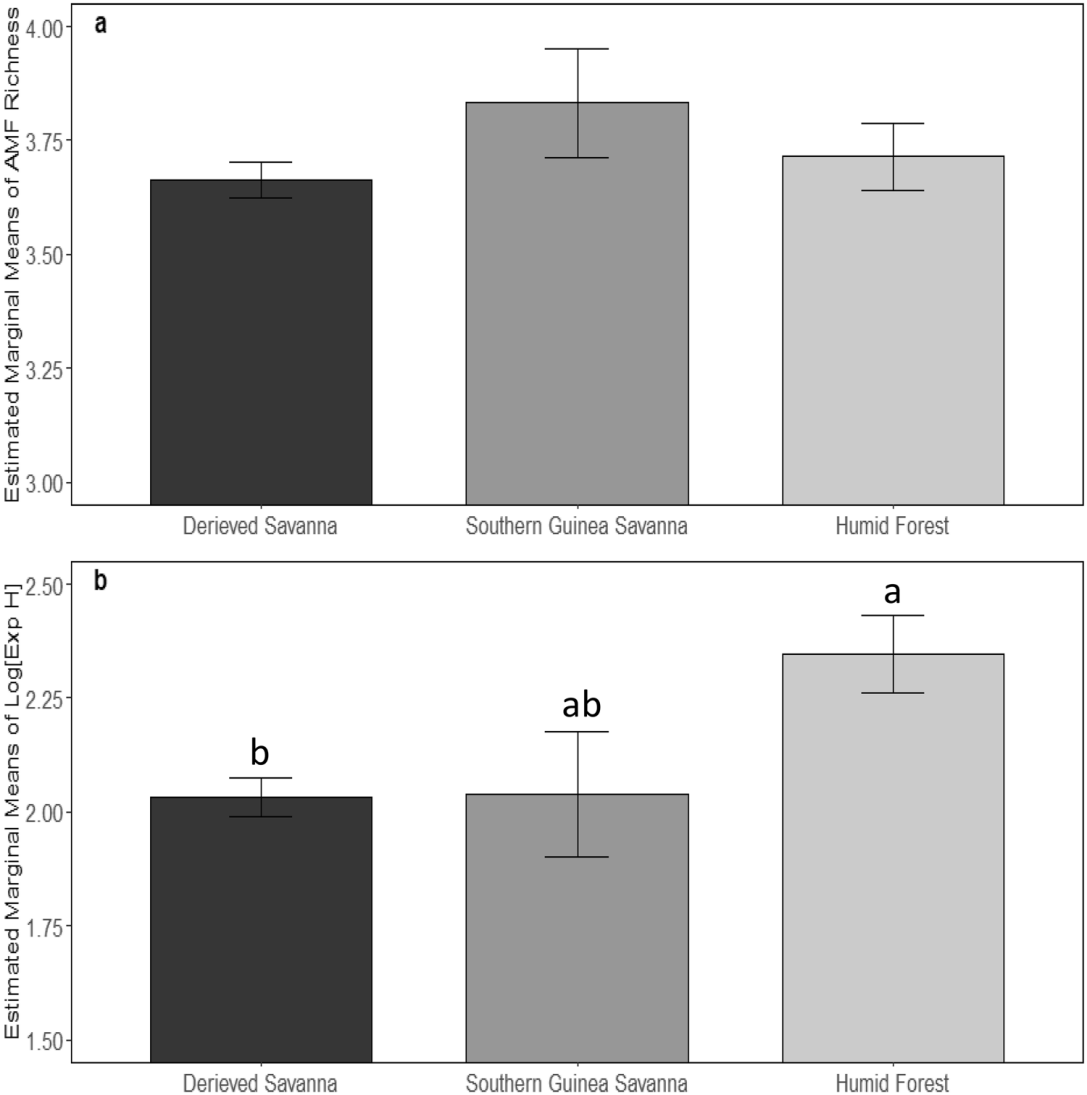
The difference in diversity indices in 60 cassava fields across three agroecology zones in Nigeria. (**a**) AMF richness, (**b**) Log (Exp H’). Bars represent estimated marginal means. Bars topped by the same letter do not differ significantly by Tukey HSD post hoc tests on GLMM and LMM models with fields as random factors

In the different agro-ecological zones, Exp(H’) differed significantly, while richness did not (Fig. 2a). The post hoc pairwise comparison showed that Exp(H’) was highest in humid forest (Fig. 2b) and differed significantly from Exp(H’) in the derived savanna zone (*p* = 0.0031) but Exp(H’) in both derived savanna and the southern Guinea savanna was not different significantly (*p* = 0.990).

### Effects of soil characteristics and management practices on AMF communities

**Fig. 3:**
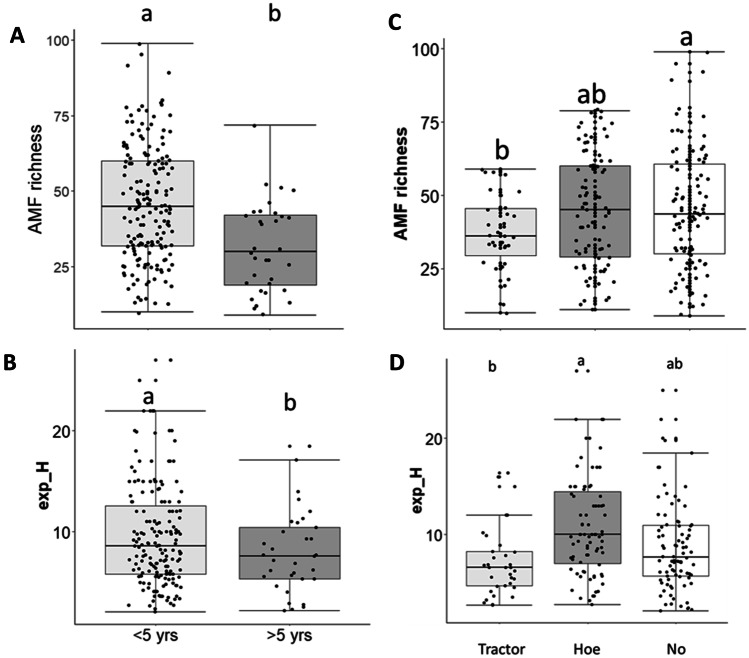
The means of AMF diversity indices in 60 cassava fields across six states in Nigeria based on some agricultural practices. (**A**) AMF richness as affected by fallow periods. (**B**) Exp H’ as affected by fallow periods. (**C**) AMF richness as affected by tillage. (**D**) Exp H’ as affected by tillage. Boxplots topped by the same letter do not differ significantly by Tukey HSD post hoc tests. The horizontal line in the middle of the box is the median value of the scores and the lower and upper boundaries indicate the 25th and 75th percentiles, respectively. The whisker presents the smallest/largest value greater/less than the lower /upper quantile minus/plus times the interquartile range where outliers fall beyond whiskers

OTU richness significantly decreased with increasing Olsen phosphorus content (Table 1); there was a highly significant negative correlation (*r* =  − 0.21, *p* = 0.0091) between OTU richness and soil Olsen P in the derived savanna zone (Fig. S4). OTU richness also increased with total soil N concentration and was low when the fallow period was longer than 5 years (Table 1) (Fig. 3a) and when tilled by ploughing with a tractor (Fig. 3c). Exp(H’) increased significantly under hoe tillage and with short fallow periods (Table 1) (Fig. 3b and d). Although the use of tractor ploughing was only typical in the derived savanna zone (Table S5), the species indicator analysis revealed preference of the identified OTUs for different soil pH ranges (Table S3) and tillage operations (Table S4). Extremely acidic soils had 33 indicator OTUs, neutral soils had 8 OTUs and slightly alkaline soils had 30 OTUs. Furthermore, under zero tillage, tractor ploughing and hoe tillage, there were 17 indicator OTUs, 23 OTUS and 34 OTUs, respectively (Table S4). Furthermore, the indicator species analysis revealed distinct affinities of AMF taxa to zero-tilled fields when compared with hoe-tilled and tractor-ploughed fields. Most families, i.e. Glomeraceae, Diversisporaceae, Acaulosporaceae, Gigasporaceae, Claroideoglomeraceae and Archaeosporaceae, were found in the hoe-tilled fields while in zero-tilled and tractor-ploughed fields the Glomeraceae predominated.

**Table 1:** Effects of soil chemical properties and management practices on AMF richness and Log(Exp(H’)). Results for AMF richness were inferred from a generalized linear mixed model with Poisson distribution, while that of Log(Exp(H’)) was inferred using a linear mixed model

	AMF richness			Log(Exp(H’))		
	Estimate + SE	*t*-Value	*p* value	Estimate + SE	*t*-Value	*p* value
(Intercept)	351.020.06	12.91	** < 0.001**	2.34 ± 0.31	7.48	** < 0.001**
Olsen P (mg kg^−1^)	− 0.17 ± 0.05	− 3.61	** < 0.001**	− 0.08 ± 0.05	− 1.59	0.11
pH	0.04 ± 0.05	0.83	0.41	− 0.03 ± 0.05	− 0.62	0.53
Total N (g kg^−1^)	0.85 ± 0.32	2.59	**0.01**	− 0.40 ± 0.38	1.05	0.29
Organic carbon (%)	− 0.64 ± 0.02	− 2.16	0.03	− 0.23 ± 0.34	− 0.68	0.49
Mounds	− 0.012 ± 0.09	− 0.15	0.89	0.01 ± 0.09	0.09	0.93
Ridge	0.01 ± 0.08	0.17	0.87	0.08 ± 0.09	0.72	0.39
Zero plough	0.08 ± 0.07	1.10	0.27	− 0.16 ± 0.08	− 1.96	**0.05**
Tractor ploughed	− 0.17 ± 0.10	− 1.67	0.09	− 0.36 ± 0.11	− 3.01	** < 0.01**
Fallow period > 5 yrs	− 0.42 ± 0.09	− 4.36	** < 0.001**	− 0.15 ± 0.11	− 1.32	**0.019**

Values are means followed by standard errors

### Identification of key factors altering AMF community composition

**Fig. 4:**
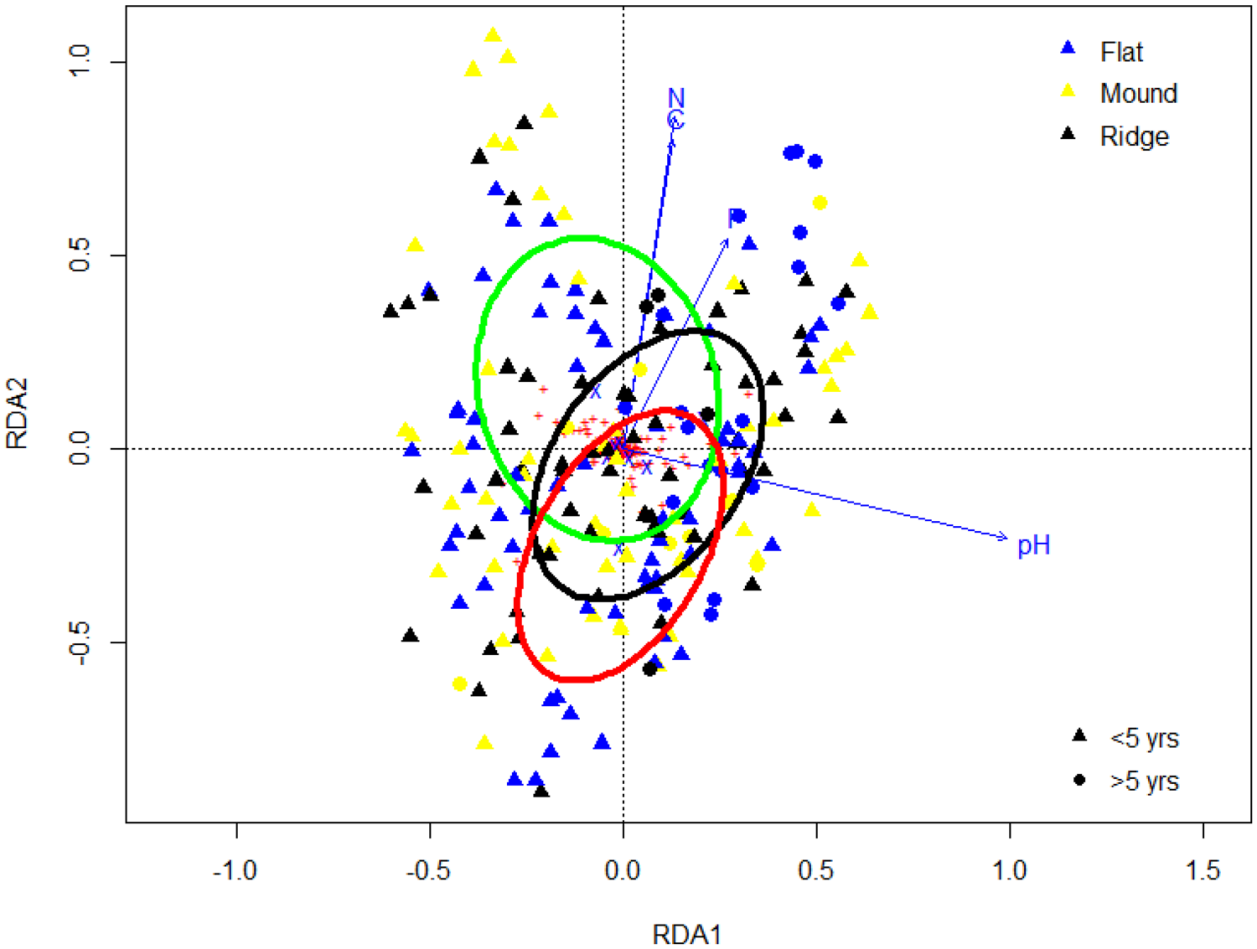
Ordination plot of the redundancy analysis (RDA) of AMF communities in the root of cassava in 60 sampling locations in three agroecology zones in Nigeria showing the effects of soil chemical properties and soil management practices on AMF composition. Arrows indicate environmental variables explaining a significant proportion of the AMF communities (as determined with forward selection, Table 2). Ellipses represent confidence regions based on SD from the centroid for management practice: green for hoe tillage, black for no-tillage and red for tractor tillage

**Fig. 5:**
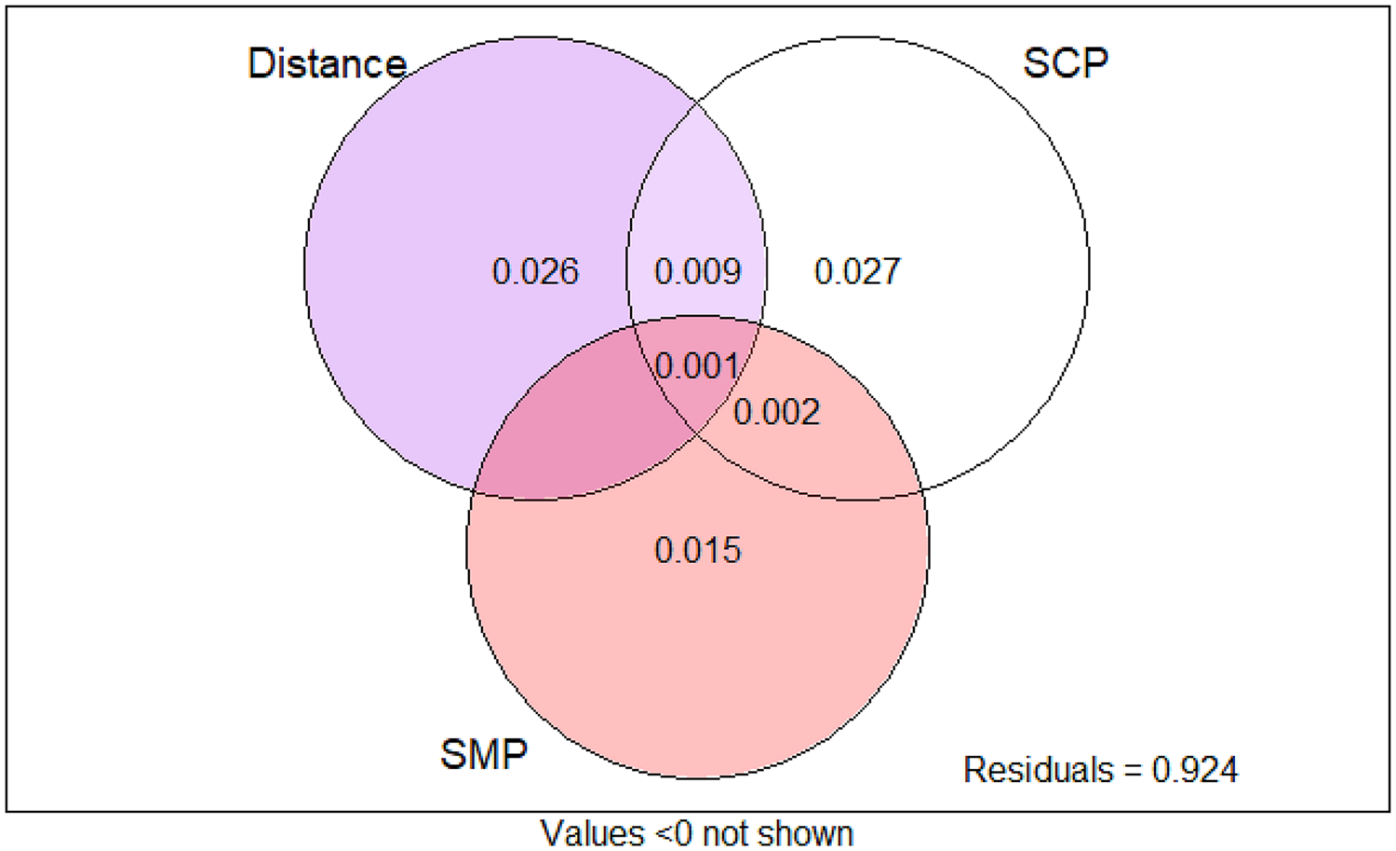
Venn diagrams depicting variance partitioning of three groups of explanatory variables, geographical distance (Distance), soil chemical properties (SCP) and soil management practices (SMPs). The overlap represents shared variation among explanatory matrices. Numbers indicate adjusted *R*^2^ values

The RDA permutation test showed that all measured soil chemical variables, pH (*F*_1,197_ = 13.17, *p* = 0.001), Olsen P (*F*_1,197_ = 3.66, *p* = 0.001), total N (*F*_1,197_ = 3.85, *p* = 0.001) and organic C (*F*_1,197_ = 1.73, *p* = 0.023), significantly affected the AMF community composition. Also the fallow length (*F*_1,197_ = 3.60, *p* = 0.001), type of tillage (*F*_2,197_ = 3.55,*p* = 0.001) and soil shaping (*F*_2,197_ = 1.79, *p* = 0.006) significantly contributed to differences in AMF community composition, with fallow length and tillage explaining most of the variance (Table 2, Fig. 4). The RDA-based variance partitioning (Fig. 5) revealed that soil chemical properties (*R*^2^ adjusted = 0.027) and geographical distance (*R*^2^ adjusted = 0.026) explained more of the variance in AMF composition than soil management practices (*R*^2^ adjusted = 0.015). The overlap between soil management practices and geographical distance (*R*^2^ adjusted = 0.009) and that of soil management practices and soil chemical properties (*R*^2^ adjusted = 0.002) contributed only minimally (*R*^2^ adjusted = 0.003).

**Table 2:** Results of the permutation tests of the canonical redundancy analysis (RDA) testing for significant relationships between AMF communities and explanatory matrices in the root of cassava in 60 sampling fields in three agro-ecological zones in Nigeria (by forward selection). Results are based on 999 permutations

	Df	Variance	*F*	Pr(> *F*)
Fallow	1	0.011	3.60	0.001
Tillage	2	0.022	3.55	0.001
Soil shaping	2	0.011	1.79	0.006
pH	1	0.041	13.17	0.001
Olsen P	1	0.011	3.66	0.001
Total N	1	0.012	3.85	0.001
Organic carbon	1	0.005	1.73	0.023

## Discussion

Despite the importance of cassava as a staple crop, little is known about the AMF communities associated with the crop. Yet, such knowledge is useful for potential future applications and enhancing the productivity and resilience of cassava. Here, we used DNA meta-barcoding to assess the variability of cassava AMF communities over a very large scale, to determine its environmental drivers and to evaluate the role of fallow length and management (including type of soil tillage and soil shaping).

The 515 OTUs detected in this study seem relatively small compared with a previous study on cassava in three agro-ecological zones in Cote d’Ivoire (Séry et al. 2018) which reported 1409 OTUs detected from soil by sequencing the LSU rRNA gene. The differences in the number of OTUs could be due to differences in the target regions of the nuclear ribosomal DNA (rDNA) and to DNA having been extracted from the soil, not from cassava roots. When relating this result to that of other crops such as yam, studies in other Western African countries have confirmed that AMF communities associated with one particular crop can differ strongly from one agro-ecological zone to another (Tchabi et al. 2008; Sarkodee-Addo et al. 2020). Similarly, the dissimilarities among AMF composition revealed in this study were further corroborated by the study of Van der Gast et al. (2011) that observed that geological characteristics (using a 250 km distance–decay analysis) significantly influenced AMF distribution in horticultural fields across England.

While we found evidence for taxonomic differences at the AMF family level, the AMF identified belonged mostly to the family *Glomeraceae*, which is consistent with the results of Peña-Venegas et al. (2019) who found that this family was dominant in cassava roots on different Amazonian soil types. The dominance of *Glomeraceae* probably is driven by the tendency of species within the family to colonize plant roots more rapidly than those of *Acaulosporaceae* and *Gigasporaceae* (Xu et al. 2017; Hempel et al. 2007). Moreover, *Glomeraceae* generally invest more in their intraradical than extraradical structures (Hart et al. 2019). Other families, *Diversisporaceae*, *Acaulosporaceae*, *Gigasporaceae* and *Claroideoglomeraceae*, were only marginally present in roots. Especially *Diversisporaceae* and *Gigasporaceae* are known to be poor root colonizers, regenerating from spores rather than from hyphal fragments (Hart et al. 2002; Hempel et al. 2007), whereas members of *Acaulosporaceae* and *Archaeosporaceae* have an affinity to acidic soils (Sieverding 1990; Straker et al. 2010; Peña-Venegas et al. 2019). An important observation of our study is that different AMF genera (other than *Glomus*) dominated each agro-ecological zone even though the soils were generally acidic. The strongly acidic humid forest soil, caused by the type of parent materials and high rainfall, exceeding 3000 mm annually (Effiong and Ibia 2009; Ojiako et al. 2018); more *Scutellospora* spp. were found than in the other AEZs. A high abundance of members of the genera *Acaulospora* and *Scutellospora* also has been reported in other acidic tropical soils (Sieverding 1990; Mathimaran et al. 2007). In the moderately acidic derived savanna zone, *Gigaspora* predominated, while the slightly acidic southern Guinea savanna soils harboured increased *Acaulospora*. Our findings indicate that a slight change in soil pH could lead to a change in the genera of AMF present, even among those belonging to the same family.

The average Olsen P concentration of all soils was only one-tenth of the critical level of 3.5–8 mg/kg (Van der Zaag et al. 1979; Howeler 1989). Yet in this study, despite the acidity of the soil and the low value of P, an increase in available P led to a decrease in AMF richness in the derived savanna zone. Usually, acidic soils with high levels of Fe and Al will fix P, keeping available P low, but a large part of the variation here is linked to a high gradient of soil phosphorus in a few fields treated with organic liquid fertilizer during the growth phase of the crop. A meta-analysis from 54 published studies conducted over the last 20 years reported that even nutrient enrichment of organic origin can reduce native AMF richness in soils relative to no fertilization (Jiang et al. 2021). This reduction is mentioned to be influenced by a shift in the balance of native AMF species (Johnson et al. 2015). While we found no effects of soil acidity on AMF richness or diversity, our RDA analysis showed that soil pH was the most important measured environmental factor driving AMF community composition. Furthermore, we found more indicator genera in the strongly acidic soils. Many previous studies have reported soil acidity to influence AMF community composition (Lekberg et al. 2008; Dumbrell et al. 2010; Bainard et al. 2014; Rincón et al. 2021), supporting the view that different AMF genera exhibited different sensitivities to soil pH. The reason for this is likely connected with the major role of soil pH in changing the physiological status of AMF, affecting sporulation (Wang et al. 1993; Guo et al. 1996) and growth of hyphae (Seguel et al. 2013). Although the variance partitioning in this study showed that soil chemical properties explained most of the variability in AMF composition, the total explained variance was low and this could be attributed to intrinsic methodological issues as described by Økland (1999).

Most low-input agricultural fields in Africa usually are left to fallow for several years to accumulate organic matter and nutrients. The time required to attain sufficient organic carbon and nutrient levels is determined by the soil type and climatic conditions (Nye and Greenland 1960), yet today fallow length mostly is determined by farmers’ land endowments. Because cassava is known to produce on little fertile soils, a majority of farmers plant the crop on fields after shortened fallow periods or no fallow at all. Although the fallow vegetation may vary within agro-ecological zones, fallows of the humid forest zones usually are dominated by woody perennial shrubs and trees. Perennial shrubs, trees and grasses dominate fallows of the derived savanna and grasses dominate fallows of the southern Guinea savanna (National Research Council 1993).

The conversion of forest to agricultural land can be expected to reduce AMF richness and diversity, and increasing fallow duration may result in increasing AMF richness following recolonization by a diversity of ruderal plant species. During fallow periods, ruderal plants may temporarily sustain active AMF propagules in the soil, which in turn promote AMF richness and diversity and which later may colonize crops and weeds during the agricultural cycle (Hausmann and Hawkes 2009; Ramos-Zapata et al. 2012). Despite these potential benefits of ruderal plants under increased length of fallow, however, the methods of removal of fallow vegetation (slash and burn or the use of herbicides) could additionally affect AMF inoculum potential (Kurle and Pfleger, 1994), richness and composition. Barraclough and Olsson (2018) reported that slashing and burning of a deforested zone led to a 27% decline in AMF root colonization. Also the use of glyphosate herbicides after slashing caused about a 20% decrease in total AMF colonization (Ramos-Zapata et al. 2012). All this may explain our observation that long fallow reduced AMF richness, which was in line with the results of De Bauw et al. (2021), yet we have no data on the methods that were used to remove fallow vegetation and how it related to the fallow period. It is important to bear in mind that the lower AMF richness detected in long fallows in this study also could be a result of under-sampling (Table S5) of long fallow periods. To be able to fully test the effects of fallow, further studies should be designed carefully in order to take into account the effect of both the fallow length as well as the type of fallow vegetation on AMF community assembly.

We found that tillage type affected AMF community composition. Tillage operations have been shown to cause shifts in AMF community composition (Alguacil et al. 2016; Jemo et al. 2018; Kabir 2011; Lekberg et al. 2008; Oehl et al. 2010). This could be explained by disruption of extraradical mycelium and dilution of AMF propagules (Sieverding 1990; Jansa et al. 2002) leading to the promotion or impairment of specific AMF groups depending on their colonizing mechanism (Abbott et al. 1994; Klironomos and Hart 2002). We found that hoe and tractor tillage operations increased AMF Shannon diversity, while zero tillage decreased Shannon diversity. The absence of an effect of tillage on richness, however, suggests that tillage affects the relative abundance of different AMF taxa, rather than their presence. Because hoe-tilled soils can be considered to have intermediate disturbance compared to zero tillage and tractor ploughing, our results may correspond to the so-called intermediate disturbance hypothesis which states that at an intermediate level of disturbance, diversity will be at its peak due to the prevention of competitive exclusion (Connell 1976; Huston 1979). However, there is a non-representation of all ploughing types in all or at least 2 agro-ecological zones, e.g. tractor ploughing was only used in the derived savanna and this could somewhat reduce the generality of the results on the effect of tractor ploughing. Unlike tillage, post tillage soil shaping (i.e. flat, ridge or mound) was little important in explaining the variation in AMF composition, probably because of the low level of soil disturbance or because prior ploughing caused intensive disturbance. Because farmers are targeting high yields, implying large bulking cassava roots, conditions permitting increases in root diameter and length of the bulking root portion are required. Onasanya et al. (2021) showed that root yields are positively affected by ploughing and ridging on fertile soils, yet less so on poor soils. Thus planting in hoe- or tractor-tilled soil has been encouraged. Although much emphasis is laid on the management practices that lead to cassava yield increase, it also is crucial to determine the role of mycorrhizal specificity and diversity on the crop’s growth, because tillage practices that promote AMF diversity and composition may not positively affect crop yield. For example, a meta-analysis by Van Geel et al. (2016), analysing results from 115 inoculation studies, including 435 experiments, showed that a high dominance of one beneficial arbuscular mycorrhizal taxon may be most beneficial to crop species under relatively controlled conditions. In this regard, considering the particular AMF taxon-host plant combination that will maximize cassava yield from the plethora of AMF taxa found in this study may help assuage any potential limitations of high AMF diversity resulting from prevailing soil management practices. On the other hand, locally changing environmental conditions may make this strategy risky.

## Conclusions

Overall, our results show that cassava roots are highly colonized and that AMF associated with cassava exhibit taxonomic differences at family and genus levels across different spatial scales. Moreover, soil chemical properties and land management practices can lead to significant shifts in AMF community composition. Soil pH played a crucial role in shaping AMF communities, while the fallow length and tillage intensity have a great influence on AMF composition and diversity. A next logical step in research is to understand what the consequences of these altered AMF communities are for cassava production under certain soil chemical properties and land management practices, and to verify whether an enhanced cassava-AMF symbiosis will result in improved productive, resilient and sustainable cassava production systems in Nigeria.

## Supplementary information

Below is the link to the electronic supplementary material.

Supplementary file1 (DOCX 494 KB)

## Data Availability

The datasets generated during and/or analysed during the current study are available from the corresponding author on reasonable request. The raw sequence data generated in this study have been deposited with links to BioProject accession number PRJNA777576 in the NCBI BioProject database (https://www.ncbi.nlm.nih.gov/bioproject/).
