# Supplementary material for: Spatial variability and environmental drivers of cassava—arbuscular mycorrhiza fungi (AMF) associations across Southern Nigeria
Source: Mycorrhiza. 2022 Jan 4;32(1):1–13. doi: 10.1007/s00572-021-01058-x (PMC8786768; doi:10.1007/s00572-021-01058-x)
Supplement: Supplementary file 1 — Supplementary file1 (DOCX 494 KB) [file 572_2021_1058_MOESM1_ESM.docx]

**Spatial variability and environmental drivers of Cassava – AMF associations across Southern Nigeria**

Bolaji Thanni^1,3^, Roel Merckx^1^, Pieterjan De Bauw^1,^ Margaux Boeraeve^2^, Gerrit Peeters^2^, Stefan Hauser ^3^, Olivier Honnay^2^

^1^ *Department of Earth and Environmental Sciences, Division Soil and Water Management, KU Leuven, Kasteelpark Arenberg 20-3001 Leuven, Belgium*

*^2^Department of Biology, Plant Conservation and Population Biology, KU Leuven, B-3001 Leuven, Belgium*

*^3^ Root and Tuber Agronomy, International Institute of Tropical Agriculture, Ibadan, Nigeria*

*Corresponding author: Bolaji Thanni, Soil and Water Management Division, Department of Earth and Environmental Science, KU Leuven, Kasteelpark Arenberg 20, box- 3001, 3001 Heverlee, Belgium. Tel: ±32465770286, E-mail: [Bolaji.thanni@kuleuven.be](mailto:Bolaji.thanni@kuleuven.be)


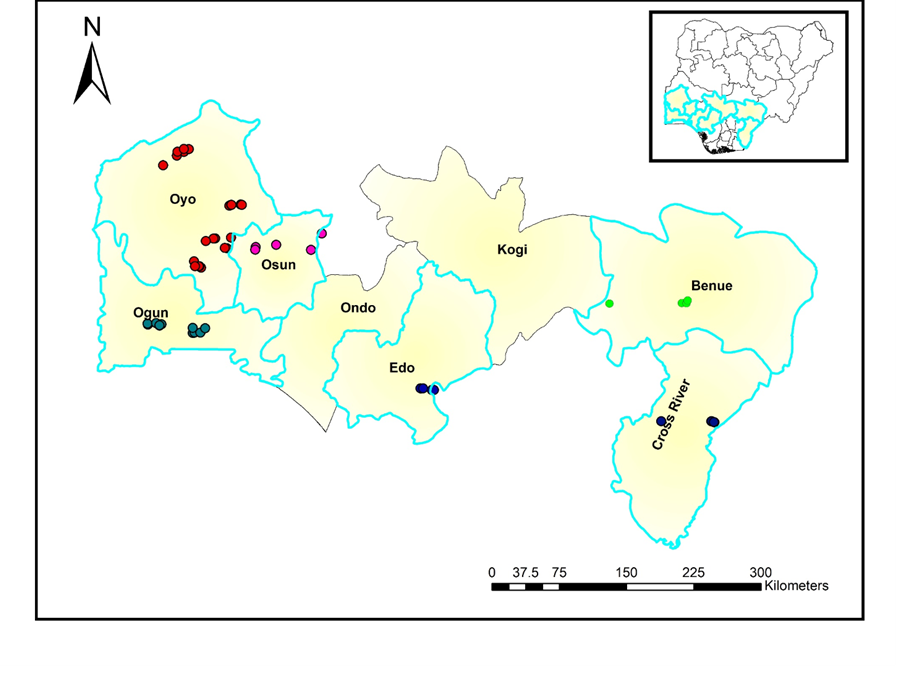


**Figure S1**. Map of the 60 sampling locations in three agroecological zones in Nigeria where cassava root and soil samples were collected.


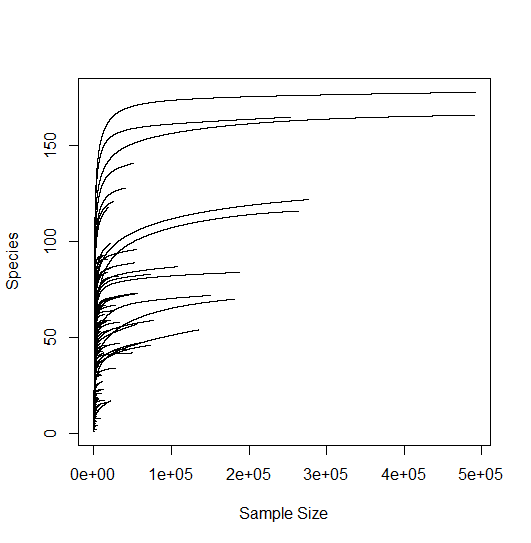

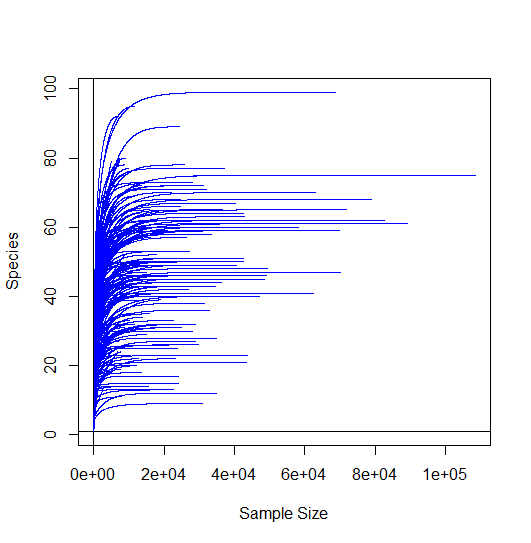


sequences

sequences

No. of OTUs

a

b

**Figure S2.** Rarefaction curves showing the number of AMF OTUs, (a) all samples (232) including insufficiently deep sequenced OTUs (b) Retained OTUs (209) after removal of the insufficiently deep sequenced samples.


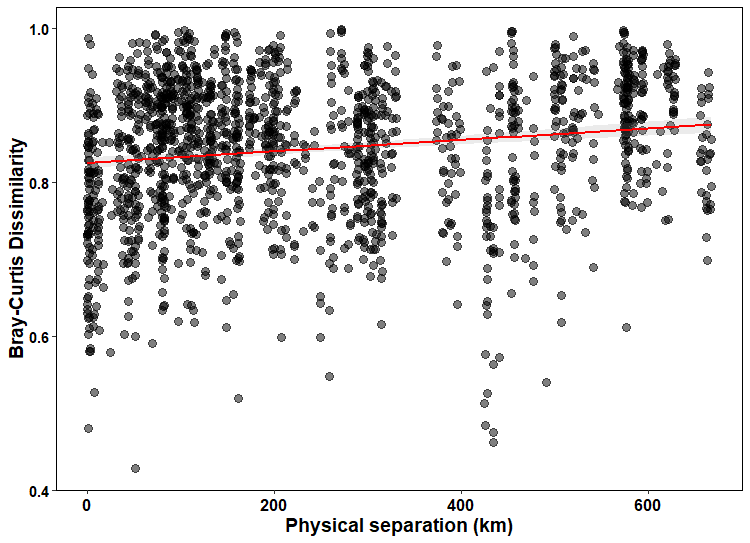


**Figure. S3**. Change in cassava AMF community dissimilarity as a function of spatial distance for 60 cassava fields in three agro-ecological zones in Nigeria. The data points show pairwise dissimilarity estimates between all fields; the bold red line shows the relationship between dissimilarity and geographical distance. There was a positive correlation between dissimilarity and distance (Mantel test) (r = 0.12, p = 0.001).


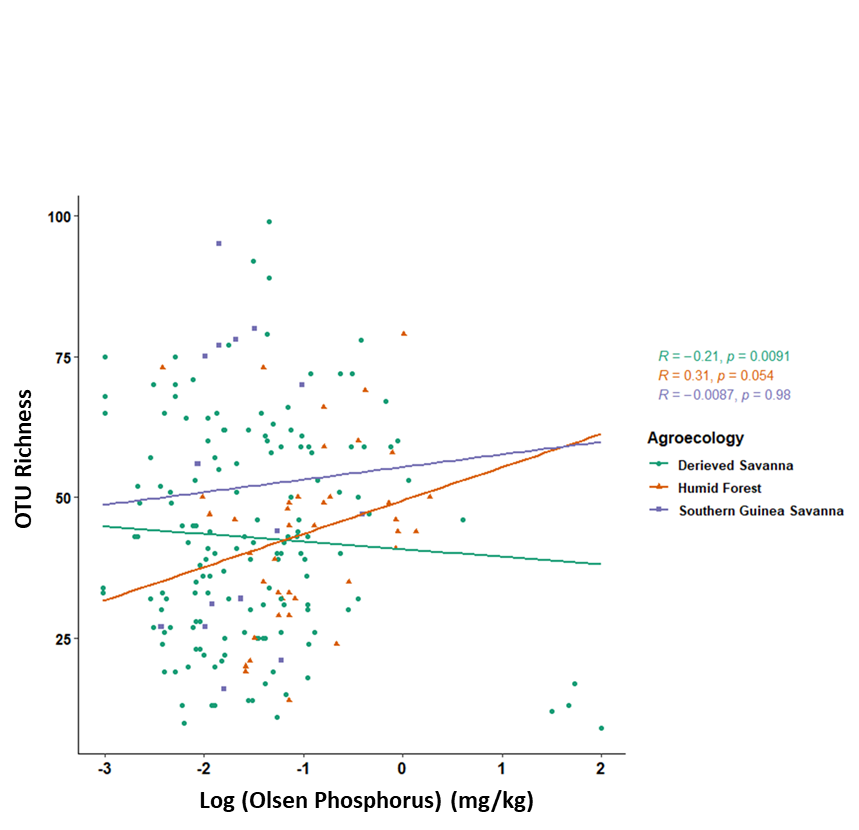


**Figure. S4:** Relation between Olsen P (Log transformed) and OTU richness for three agroecological zones in Nigeria where cassava roots and soil samples were collected. Fitted lines are presented with R = Pearson correlation coefficient.

**Table S1**: Soil Management practices and cassava varieties cultivated in the three agroecological zones in Nigeria where cassava roots and soil samples were collected.

| AEZ^†^ | State | LGA | Land clearing | Clearing of vegetation | Land preparation | Cultivars planted |
| --- | --- | --- | --- | --- | --- | --- |
| SGS | Benue | Okpokwu | Manual slashing (75%) | Slash, burn and remove  Slash and burn | Ridges and mounds | Wonana |
|  |  |  | Herbicides (25%) |  |  |  |
| DS | Ogun | Ewekoro | Manual slashing (83%) | Slash and incorporate | Flat, mounds and ridges | TMS 98/0581 |
|  |  | Ikenne | Manual slashing and herbicides (17%) | Slash and burn |  | TME 419 |
|  |  | Sagamu |  | Slash and remove |  | Sammy |
|  |  |  |  | Slash, burn and remove |  | Oko Iyawo |
|  | Oyo | AfijioAfijio, Ori Ire | Manual slashing (79%) | Slash and remove | Flat, mounds and ridges | Odongbo |
|  |  | Ido, | Mechanical slashing (17%) | Slash and incorporate |  | TMS 30572 |
|  |  | Saki West, | Herbicides (4%) |  |  | Pan rubber |
|  |  | Orire |  |  |  |  |
|  |  |  |  |  |  | TME 419 |
|  |  |  |  |  |  | Arubielu |
|  |  |  |  |  |  | Olekanaga |
|  |  |  |  |  |  | Egedudu |
|  | Osun | Obokun | Manual slashing (50%) | Slash and incorporate | Flat, mounds and ridges | TME 419 |
|  |  | Ede north | Mechanical slashing (33%) |  |  | TMS 01/1368 |
|  |  | Olaoluwa | Herbicides (17%) |  |  | TMS 98/0581 |
|  |  |  |  |  |  |  |
| HF | Edo |  | Manual slashing (100%) | Slash and incorporate | Ridges and flat | Give me chance |
|  |  | Nwaode |  | Slash and burn |  | Canopy |
|  | Cross rivers | Ikom | Manual slashing (83%) | Slash and incorporate | Ridges and mounds | TMS 01/1368 |
|  |  |  | Herbicides (17%) | Slash and remove |  | NR 8082 |
|  |  |  |  | Slash and burn |  | Canopy |
|  |  |  |  |  |  | TME 419 |

† AEZ: Agroecological zone (HF: Humid forest, SGS: Southern Guinea savanna, DS: Derived savanna

**Table S2.** Soil chemical properties and No. of OTUs in the three agroecological zones in Nigeria where cassava root and soil samples were collected. Values are means followed by the standard error. Means with the same letter do not differ significanty (P < 0.05) according to the Tukey test.

| Agroecological zones |  |  |  |  |  |
| --- | --- | --- | --- | --- | --- |
|  | pH | Olsen P  (mg kg^-1^ soil) | Total Nitrogen  (g kg^-1^ soil) | Organic carbon  (%) | No. of OTUs |
| Derieved Savanna | 6.05±0.08^c^ | 0.39±0.06^a^ | 0.65±0.03^a^ | 0.78±0.04^a^ | 38.6±1.54^a^ |
| Southern Guinea Savanna | 5.18±0.09^b^ | 0.36±0.15^a^ | 0.74±0.08^ab^ | 0.87±0.08^a^ | 41.1±3.03^a^ |
| Humid Forest | 4.80±0.04^a^ | 0.46±0.12^a^ | 0.99±0.06^b^ | 1.16±0.07^b^ | 44.6±3.86^a^ |
|  |  |  |  |  |  |

**Table S3**: Indicator operational taxonomic units (OTUs) identified for different pH ranges.

| pH | OTU_ID | Description | Family | Genus | Indicator  Value | P |
| --- | --- | --- | --- | --- | --- | --- |
| Strongly acidic | Otu21 | Gigasporaceae Scutellospora sp. VTX00041 | Gigasporaceae | Scutellospora | 0.54 | 0.0001 *** |
|  | Otu42 | Gigasporaceae Scutellospora heterogama VTX00255 | Gigasporaceae | Scutellospora | 0.515 | 0.0001 *** |
|  | Otu78 | Gigasporaceae Scutellospora LH-Sc01 | Gigasporaceae | Scutellospora | 0.384 | 0.0002 *** |
|  | Otu128 | Gigasporaceae Scutellospora sp. VTX00318 | Gigasporaceae | Scutellospora | 0.379 | 0.0002 *** |
|  | Otu192 | Glomeraceae Glomus sp. VTX00093 | Glomeraceae | Glomus | 0.377 | 0.0007 *** |
|  | Otu65 | Gigasporaceae Scutellospora sp. VTX00041 | Gigasporaceae | Scutellospora | 0.373 | 0.0006 *** |
|  | Otu172 | Gigasporaceae Scutellospora sp. VTX00318 | Gigasporaceae | Scutellospora | 0.364 | 0.0007 *** |
|  | Otu49 | Gigasporaceae Gigaspora sp. | Gigasporaceae |  | 0.362 | 0.0007 *** |
|  | Otu84 | Glomeraceae Glomus sp. VTX00096 | Glomeraceae | Glomus | 0.357 | 0.0007 *** |
|  | Otu86 | Glomeraceae Glomus sp. VTX00222 | Glomeraceae | Glomus | 0.357 | 0.0012 ** |
| Otu39 | Acaulosporaceae Acaulospora sp. | Acaulosporaceae | Acaulospora | 0.345 | 0.0003 *** |  |
|  | Otu1116 | Glomeraceae Glomus sp. VTX00204 | Glomeraceae | Glomus | 0.328 | 0.0025 ** |
|  | Otu109 | Glomeraceae Glomus sp. VTX00167 | Glomeraceae | Glomus | 0.298 | 0.0033 ** |
|  | Otu56 | Glomeraceae Glomus sp. VTX00410 | Glomeraceae | Glomus | 0.29 | 0.0057 ** |
|  | Otu115 | Glomeraceae Glomus sp. VTX00126 | Glomeraceae | Glomus | 0.289 | 0.0032 ** |
|  | Otu1246 | Gigasporaceae Gigaspora NF32 VTX00039 | Gigasporaceae | Gigaspora | 0.285 | 0.0059 ** |
|  | Otu227 |  |  |  | 0.282 | 0.0054 ** |
|  | Otu22 | Glomeraceae Glomus sp. VTX00420 | Glomeraceae | Glomus | 0.272 | 0.0145 * |
|  | Otu270 | Paraglomeraceae Paraglomus sp. VTX00375 | Paraglomeraceae | Paraglomus | 0.258 | 0.0099 ** |
|  | Otu361 | Glomeraceae Glomus sp. VTX00130 | Glomeraceae | Glomus | 0.258 | 0.0071 ** |
|  | Otu33 | Glomeraceae Glomus MO-G44 VTX00410 | Glomeraceae | Glomus | 0.258 | 0.0156 * |
|  | Otu223 | Glomeraceae Glomus sp. VTX00077 | Glomeraceae | Glomus | 0.253 | 0.0207 * |
|  | Otu108 | Glomeraceae Glomus Yoshimura13a Glo7 | Glomeraceae | Glomus | 0.25 | 0.0119 * |
|  | Otu60 | Glomeraceae Glomus sp. | Glomeraceae | Glomus | 0.247 | 0.0196 * |
|  | Otu23 | Glomeraceae Glomus sp. VTX00167 | Glomeraceae | Glomus | 0.232 | 0.0209 * |
|  | Otu155 | Glomeraceae Glomus sp. VTX00368 | Glomeraceae | Glomus | 0.228 | 0.0166 * |
|  | Otu197 | Glomeraceae Glomus sp. VTX00089 | Glomeraceae | Glomus | 0.226 | 0.0201 * |
|  | Otu740 | Glomeraceae Glomus sp. VTX00222 | Glomeraceae | Glomus | 0.225 | 0.0154 * |
|  | Otu150 | Claroideoglomeraceae Claroideoglomus Mueller15 otu9 | Claroideoglomeraceae | Claroideoglomus | 0.225 | 0.0136 * |
|  | Otu168 | Glomeraceae Glomus sp. VTX00089 | Glomeraceae | Glomus | 0.225 | 0.0133 * |
|  | Otu169 | Glomeraceae Glomus sp. VTX00369 | Glomeraceae | Glomus | 0.224 | 0.0198 * |
|  | Otu329 | Glomeraceae Glomus sp. | Glomeraceae | Glomus | 0.217 | 0.0268 * |
|  | Otu291 | Glomeraceae Glomus sp. VTX00167 | Glomeraceae | Glomus | 0.215 | 0.0126 * |
|  | Otu63 | Glomeraceae Glomus sp. VTX00400 | Glomeraceae | Glomus | 0.214 | 0.0334 * |
|  | Otu175 | Acaulosporaceae Acaulospora Franke B VTX00026 | Acaulosporaceae | Acaulospora | 0.214 | 0.0248 * |
|  | Otu67 | Claroideoglomeraceae Claroideoglomus sp. VTX00278 | Claroideoglomeraceae | Claroideoglomus | 0.214 | 0.0235 * |
|  | Otu761 | Glomeraceae Glomus sp. VTX00312 | Glomeraceae | Glomus | 0.211 | 0.0280 * |
|  | Otu165 | Glomeraceae Glomus sp. VTX00167 | Glomeraceae | Glomus | 0.21 | 0.0203 * |
|  | Otu1036 | Gigasporaceae Scutellospora sp. | Gigasporaceae | Scutellospora | 0.208 | 0.0463 * |
|  | Otu72 | Glomeraceae Glomus sp. VTX00120 | Glomeraceae | Glomus | 0.207 | 0.0337 * |
|  | Otu773 | Glomeraceae Glomus sp. VTX00222 | Glomeraceae | Glomus | 0.206 | 0.0329 * |
|  | Otu850 | Glomeraceae Glomus sp. VTX00096 | Glomeraceae | Glomus | 0.206 | 0.0319 * |
|  | Otu47 | Glomeraceae Glomus sp. VTX00268 | Glomeraceae | Glomus | 0.204 | 0.0453 * |
|  | Otu164 | Archaeosporaceae Archaeospora sp. VTX00051 | Archaeosporaceae | Archaeospora | 0.203 | 0.0381 * |
|  | Otu686 | Glomeraceae Glomus sp. | Glomeraceae | Glomus | 0.203 | 0.0153 * |
|  | Otu247 | Archaeosporaceae Archaeospora sp. VTX00051 | Archaeosporaceae | Archaeospora | 0.198 | 0.0445 * |
|  |  |  |  |  |  |  |
| Moderately acidic | Otu13 | Glomeraceae Glomus sp. | Glomeraceae | Glomus | 0.292 | 0.0089 ** |
|  | Otu865 | Gigasporaceae Scutellospora sp. VTX00041 | Gigasporaceae | Scutellospora | 0.288 | 0.0089 ** |
|  | Otu12 | Glomeraceae Glomus sp. VTX00113 | Glomeraceae | Glomus | 0.272 | 0.0136 * |
|  | Otu19 | Diversisporaceae Diversispora sp. VTX00063 | Diversisporaceae | Diversispora | 0.271 | 0.0091 ** |
|  | Otu259 | Glomeraceae Glomus sp. VTX00114 | Glomeraceae | Glomus | 0.255 | 0.0170 * |
|  | Otu287 | Glomeraceae Glomus sp. VTX00080 | Glomeraceae | Glomus | 0.245 | 0.0207 * |
|  | Otu1 | Glomeraceae Glomus sp. VTX00132 | Glomeraceae | Glomus | 0.237 | 0.0337 * |
|  | Otu20 | Glomeraceae Glomus sp. VTX00167 | Glomeraceae | Glomus | 0.237 | 0.0246 * |
|  | Otu383 | Glomeraceae Glomus sp. | Glomeraceae | Glomus | 0.236 | 0.0219 * |
|  | Otu471 | Glomeraceae Glomus sp. VTX00185 | Glomeraceae | Glomus | 0.218 | 0.0459 * |
|  | Otu28 | Glomeraceae Glomus sp. VTX00418 | Glomeraceae | Glomus | 0.206 | 0.0387 * |
|  | Otu885 | Glomeraceae Glomus sp. VTX00069 | Glomeraceae | Glomus | 0.206 | 0.0434 * |
|  | Otu7 | Glomeraceae Glomus Yamato09 E VTX00063 | Glomeraceae | Glomus | 0.198 | 0.0490 * |
|  |  |  |  |  |  |  |
| Slightly acidic | Otu102 | Glomeraceae Glomus sp. VTX00111 | Glomeraceae | Glomus | 0.385 | 0.0001 *** |
|  | Otu337 | Glomeraceae Glomus sp. VTX00092 | Glomeraceae | Glomus | 0.374 | 0.0001 *** |
|  | Otu1098 | Glomeraceae Glomus sp. VTX00092 | Glomeraceae | Glomus | 0.35 | 0.0001 *** |
|  | Otu377 | Claroideoglomeraceae Claroideoglomus sp. VTX00341 | Claroideoglomeraceae | Claroideoglomus | 0.307 | 0.0001 *** |
|  | Otu926 | Glomeraceae Glomus sp. VTX00111 | Glomeraceae | Glomus | 0.299 | 0.0046 ** |
|  | Otu373 | Glomeraceae Glomus sp. VTX00222 | Glomeraceae | Glomus | 0.298 | 0.0019 ** |
|  | Otu641 | Glomeraceae Glomus sp. | Glomeraceae | Glomus | 0.29 | 0.0002 *** |
|  | Otu903 | Glomeraceae Glomus sp. VTX00092 | Glomeraceae | Glomus | 0.267 | 0.0052 ** |
|  | Otu364 | Glomeraceae Glomus sp. VTX00132 | Glomeraceae | Glomus | 0.24 | 0.0046 ** |
|  | Otu332 | Glomeraceae Glomus Yoshimura13a Glo1 | Glomeraceae | Glomus | 0.23 | 0.0237 * |
|  | Otu749 | Glomeraceae Glomus sp. VTX00163 | Glomeraceae | Glomus | 0.23 | 0.0210 * |
|  | Otu159 | Glomeraceae Glomus sp. VTX00103 | Glomeraceae | Glomus | 0.221 | 0.0291 * |
|  | Otu1168 | Glomeraceae Glomus sp. | Glomeraceae | Glomus | 0.193 | 0.0500 * |

Significance levels obtained by Monte Carlo Permutation tests, their abundance and taxonomic affiliation are listed

**Table S4**. Indicator operational taxonomic units (OTUs) identified for different tillage operations.

| Tillage | OTU_ID | Description | | | Family | | Genus | | Indicator  Value | P | |  |  |
| --- | --- | --- | --- | --- | --- | --- | --- | --- | --- | --- | --- | --- | --- |
| **Zero till** | Otu10 | Glomeraceae Glomus sp. | | | Glomeraceae | | Glomus | | 0.22 | 0.0044 ** | |  |  |
|  | Otu834 | Glomeraceae Glomus sp. | | | Glomeraceae | | Glomus | | 0.214 | 0.0080 ** | |  |  |
|  | Otu173 | Glomeraceae Glomus sp. VTX00092 | | | Glomeraceae | | Glomus | | 0.201 | 0.0059 ** | |  |  |
|  | Otu110 | Glomeraceae Glomus sp. VTX00092 | | | Glomeraceae | | Glomus | | 0.196 | 0.0184 * | |  |  |
|  | Otu147 | Glomeraceae Glomus sp. VTX00120 | | | Glomeraceae | | Glomus | | 0.184 | 0.0118 * | |  |  |
|  | Otu121 | Glomeraceae Glomus sp. VTX00209 | | | Glomeraceae | | Glomus | | 0.183 | 0.0196 * | |  |  |
|  | Otu749 | Glomeraceae Glomus sp. VTX00163 | | | Glomeraceae | | Glomus | | 0.183 | 0.0175 * | |  |  |
|  | Otu63 | Glomeraceae Glomus sp. VTX00400 | | | Glomeraceae | | Glomus | | 0.183 | 0.0176 * | |  |  |
|  | Otu156 | Glomeraceae Glomus sp. VTX00199 | | | Glomeraceae | | Glomus | | 0.182 | 0.0191 * | |  |  |
|  | Otu1074 | Claroideoglomeraceae | | | Claroideoglomus | | Claroideoglomus | | 0.181 | 0.0311 * | |  |  |
|  | Otu186 | Glomeraceae Glomus sp. VTX00454 | | | Glomeraceae | | Glomus | | 0.178 | 0.0232 * | |  |  |
|  | Otu628 | Glomeraceae Glomus sp. | | | Glomeraceae | | Glomus | | 0.172 | 0.0491 * | |  |  |
|  | Otu288 | Glomeraceae Glomus sp. VTX00080 | | | Glomeraceae | | Glomus | | 0.169 | 0.0432 * | |  |  |
|  | Otu918 | Glomeraceae Glomus Franke A VTX00204 | | | Glomeraceae | | Glomus | | 0.167 | 0.0346 * | |  |  |
|  | Otu168 | Glomeraceae Glomus sp. VTX00089 | | | Glomeraceae | | Glomus | | 0.152 | 0.0493 * | |  |  |
|  | Otu1098 | Glomeraceae Glomus sp. VTX00092 | | | Glomeraceae | | Glomus | | 0.148 | 0.0492 * | |  |  |
|  | Otu436 | Glomeraceae Glomus sp. VTX00146 | | | Glomeraceae | | Glomus | | 0.144 | 0.0350 * | |  |  |
|  | Otu10 | Glomeraceae Glomus sp. | | | Glomeraceae | | Glomus | | 0.22 | 0.0044 ** | |  |  |
|  | Otu834 | Glomeraceae Glomus sp. | | | Glomeraceae | | Glomus | | 0.214 | 0.0080 ** | |  |  |
|  | Otu173 | Glomeraceae Glomus sp. VTX00092 | | | Glomeraceae | | Glomus | | 0.201 | 0.0059 ** | |  |  |
|  | Otu110 | Glomeraceae Glomus sp. VTX00092 | | | Glomeraceae | | Glomus | | 0.196 | 0.0184 * | |  |  |
|  | Otu147 | Glomeraceae Glomus sp. VTX00120 | | | Glomeraceae | | Glomus | | 0.184 | 0.0118 * | |  |  |
|  | Otu121 | Glomeraceae Glomus sp. VTX00209 | | | Glomeraceae | | Glomus | | 0.183 | 0.0196 * | |  |  |
|  | Otu749 | Glomeraceae Glomus sp. VTX00163 | | | Glomeraceae | | Glomus | | 0.183 | 0.0175 * | |  |  |
|  | Otu63 | Glomeraceae Glomus sp. VTX00400 | | | Glomeraceae | | Glomus | | 0.183 | 0.0176 * | |  |  |
|  |  |  | | |  | |  | | 0.306 | 0.0002 *** | |  |  |
| **Tractor plough** | Otu432 | Glomeraceae Glomus sp. VTX00092 | | | Glomeraceae | | Glomus | | 0.295 | 0.0001 *** | |  |  |
|  | Otu1278 | Glomus sp. clone sp44 small subunit ribosomal RNA gene, partial sequence | | | Glomeraceae | | Glomus | | 0.285 | 0.0001 *** | |  |  |
|  | Otu88 | Glomeraceae Glomus sp. VTX00063 | | | Glomeraceae | | Glomus | | 0.279 | 0.0001 *** | |  |  |
|  | Otu38 | Glomeraceae Glomus sp. | | | Glomeraceae | | Glomus | | 0.268 | 0.0003 *** | |  |  |
|  | Otu25 | Glomeraceae Glomus sp. | | | Glomeraceae | | Glomus | | 0.259 | 0.0001 *** | |  |  |
|  | Otu968 | Glomeraceae Glomus sp. | | | Glomeraceae | | Glomus | | 0.244 | 0.0027 ** | |  |  |
|  | Otu791 | Glomeraceae Glomus sp. VTX00312 | | | Glomeraceae | | Glomus | | 0.24 | 0.0022 ** | |  |  |
|  | Otu316 | Glomeraceae Glomus sp. | | | Glomeraceae | | Glomus | | 0.237 | 0.0001 *** | |  |  |
|  | Otu1294 | Glomeraceae Glomus sp. | | | Glomeraceae | | Glomus | | 0.21 | 0.0061 ** | |  |  |
|  | Otu643 | Glomeraceae Glomus sp. VTX00113 | | | Glomeraceae | | Glomus | | 0.196 | 0.0274 * | |  |  |
|  | Otu876 | Glomus viscosum partial 18S rRNA gene, isolate EEZ20 | | | Glomeraceae | | Glomus | | 0.192 | 0.0005 *** | |  |  |
|  | Otu46 | Glomeraceae Glomus sp. VTX00419 | | | Glomeraceae | | Glomus | | 0.191 | 0.0173 * | |  |  |
|  | Otu696 | Glomeraceae Glomus sp. VTX00280 | | | Glomeraceae | | Glomus | | 0.189 | 0.0274 * | |  |  |
|  | Otu1150 | Glomeraceae Glomus sp. VTX00109 | | | Glomeraceae | | Glomus | | 0.185 | 0.0120 * | |  |  |
|  | Otu17 | Glomeraceae Glomus Glo45 VTX00109 | | | Glomeraceae | | Glomus | | 0.178 | 0.0306 * | |  |  |
|  | Otu416 | Glomeraceae Glomus Porras-Alfaro03 OTU3 VTX00418 | | | Glomeraceae | | Glomus | | 0.176 | 0.0247 * | |  |  |
|  | Otu1320 | Rhizophagus intraradices clone 5_2 18S ribosomal RNA gene, partial sequence | | | Glomeraceae | | Rhizophagus | | 0.166 | 0.0279 * | |  |  |
|  | Otu1183 | Glomus sp. Glo55 partial 18S rRNA gene, clone 14-30 | | | Glomeraceae | | Glomus | | 0.162 | 0.0459 * | |  |  |
|  | Otu581 | Diversisporaceae Diversispora sp. | | | Diversisporaceae | | Diversispora | | 0.158 | 0.0279 * | |  |  |
|  | Otu991 | Glomus sp. clone VT44 small subunit ribosomal RNA gene, partial sequence | | | Glomeraceae | | Glomus | | 0.155 | 0.0269 * | |  |  |
|  | Otu1152 | Diversisporaceae Diversispora sp. | | | Diversisporaceae | | Diversispora | | 0.148 | 0.0367 * | |  |  |
|  | Otu35 | Glomeraceae Glomus sp. VTX00067 | | | Glomeraceae | | Glomus | | 0.142 | 0.0478 * | |  |  |
|  | Otu284 | Glomeraceae Glomus sp. | | | Glomeraceae | | Glomus | | 0.306 | 0.0002 *** | |  |  |
|  | Otu432 | Glomeraceae Glomus sp. VTX00092 | | | Glomeraceae | | Glomus | | 0.295 | 0.0001 *** | |  |  |
|  | Otu1278 | Glomus sp. clone sp44 small subunit ribosomal RNA gene, partial sequence | | | Glomeraceae | | Glomus | | 0.285 | 0.0001 *** | |  |  |
|  |  |  | | |  | |  | |  |  | |  |  |
| **Hoe plough** | Otu192 | Glomeraceae Glomus sp. VTX00093 | | | Glomeraceae | | Glomus | | 0.325 | 0.0001 *** | |  |  |
|  | Otu30 | Diversisporaceae Diversispora sp. VTX00353 | | | Diversisporaceae | | Diversispora | | 0.291 | 0.0006 *** | |  |  |
|  | Otu42 | Gigasporaceae Scutellospora heterogama VTX00255 | | | Gigasporaceae | | Scutellospora | | 0.266 | 0.0009 *** | |  |  |
|  | Otu272 | Glomeraceae Glomus sp. VTX00167 | | | Glomeraceae | | Glomus | | 0.253 | 0.0010 *** | |  |  |
|  | Otu264 | Glomeraceae Glomus sp. | | | Glomeraceae | | Glomus | | 0.231 | 0.0022 ** | |  |  |
|  | Otu109 | Glomeraceae Glomus sp. VTX00167 | | | Glomeraceae | | Glomus | | 0.214 | 0.0060 ** | |  |  |
|  | Otu218 | Glomeraceae Glomus sp. | | | Glomeraceae | | Glomus | | 0.212 | 0.0099 ** | |  |  |
|  | Otu39 | Acaulosporaceae Acaulospora sp. | | | Acaulosporaceae | | Acaulospora | | 0.209 | 0.0046 ** | |  |  |
|  | Otu164 | Archaeosporaceae Archaeospora sp. VTX00051 | | | Archaeosporaceae | | Archaeospora | | 0.2 | 0.0037 ** | |  |  |
|  | Otu49 | Gigasporaceae Gigaspora sp. | | | Gigasporaceae | | Gigaspora | | 0.194 | 0.0183 * | |  |  |
|  | Otu23 | Glomeraceae Glomus sp. VTX00167 | | | Glomeraceae | | Glomus | | 0.194 | 0.0117 * | |  |  |
|  | Otu50 | Acaulosporaceae Acaulospora sp. | | | Acaulosporaceae | | Acaulospora | | 0.192 | 0.0231 * | |  |  |
|  | Otu44 | Claroideoglomeraceae Claroideoglomus sp. | | | Claroideoglomeraceae | | Claroideoglomus | | 0.189 | 0.0207 * | |  |  |
|  | Otu104 | Glomeraceae Glomus sp. VTX00360 | | | Glomeraceae | | Glomus | | 0.186 | 0.0106 * | |  |  |
|  | Otu502 | Diversisporaceae Diversispora MO-D2 VTX00353 | | | Diversisporaceae | | Diversispora | | 0.185 | 0.0283 * | |  |  |
|  | Otu54 | Glomus viscosum partial 18S rRNA gene, isolate EEZ34 | | | Glomeraceae | | Glomus | | 0.185 | 0.0269 * | |  |  |
|  | Otu543 | Glomeraceae Glomus sp. | | | Glomeraceae | | Glomus | | 0.184 | 0.0120 * | |  |  |
|  | Otu621 | Glomeraceae Glomus sp. VTX00167 | | | Glomeraceae | | Glomus | | 0.182 | 0.0215 * | |  |  |
|  | Otu992 | Glomeraceae Glomus sp. VTX00419 | | | Glomeraceae | | Glomus | | 0.178 | 0.0238 * | |  |  |
|  | Otu1224 | Acaulosporaceae Acaulospora sp. VTX00026 | | | Acaulosporaceae | | Acaulospora | | 0.177 | 0.0396 * | |  |  |
|  | Otu361 | Glomeraceae Glomus sp. VTX00130 | | | Glomeraceae | | Glomus | | 0.177 | 0.0147 * | |  |  |
|  | Otu78 | Gigasporaceae Scutellospora LH-Sc01 | | | Gigasporaceae | | Scutellospora | | 0.176 | 0.0352 * | |  |  |
|  | Otu223 | Glomeraceae Glomus sp. VTX00077 | | | Glomeraceae | | Glomus | | 0.174 | 0.0371 * | |  |  |
|  | Otu108 | Glomeraceae Glomus Yoshimura13a Glo7 | | | Glomeraceae | | Glomus | | 0.172 | 0.0175 * | |  |  |
|  |  | |  |  | |  | |  | | |  | |  |

Significance levels obtained by Monte Carlo Permutation tests, their abundance and taxonomic affiliation are listed

**Table S5**: Sampling distribution based on agricultural practices in the three agroecological zones in Nigeria where cassava root and soil samples were collected. Numbers present Cassava individuals that were sampled.

| Site characteristics | Humid  Forest | Derived  Savanna | Southern  Guinea  Savanna | Total |
| --- | --- | --- | --- | --- |
| Fallow > 5 years | 0 | 29 | 4 | 33 |
| Fallow < 5 years | 40 | 124 | 11 | 175 |
| Tractor ploughed | 0 | 35 | 0 | 35 |
| Hoe tilled | 33 | 50 | 0 | 83 |
| Zero tilled | 7 | 68 | 15 | 90 |
| Ridged | 7 | 53 | 3 | 63 |
| Flat | 18 | 64 | 0 | 82 |
| Mounds | 15 | 36 | 12 | 63 |
|  |  |  |  |  |
